# Changes in life history traits and transcriptional regulation of Coccinellini ladybirds in using alternative prey

**DOI:** 10.1186/s12864-020-6452-0

**Published:** 2020-01-14

**Authors:** Mei-Lan Chen, Yu-Hao Huang, Bo-Yuan Qiu, Pei-Tao Chen, Xue-Yong Du, Hao-Sen Li, Hong Pang

**Affiliations:** State Key Laboratory of Biocontrol, School of Life Sciences/School of Ecology, Sun Yat-sen University, Guangzhou, 510275 China

**Keywords:** Generalist ladybird, Alternative prey, Life history traits, Transcriptome

## Abstract

**Background:**

Ladybird beetles (Coleoptera, Coccinellidae) are highly diverse in their feeding habits. Most of them are specialist feeders, while some can have a broad spectrum of prey. As a representative group of generalists, the tribe Coccinellini includes many aphidophagous species, but members of this tribe also feed on other hemipterous insects including coccids, psyllids and whiteflies. As a result, several species are effective biological control agents or invasive species with serious non-target effects. Despite their economic importance, relatively little is known about how they adapt to new prey.

**Results:**

In this study, comparisons of the life history traits and transcriptomes of ladybirds fed initial (aphids) and alternative prey (mealybugs) were performed in three Coccinellini species. The use of alternative prey greatly decreased performance, implied by the significantly prolonged development time and decreased survival rate and adult weight. Prey shifts resulted in a set of differentially expressed genes encoding chemosensory proteins and digestive and detoxifying enzymes.

**Conclusions:**

Our results suggest that these generalists do not perform well when they use alternative prey as the sole nutrition source. Although their capacity for predation might have created an opportunity to use varied prey, they must adapt to physiological obstacles including chemosensing, digestion and detoxification in response to a prey shift. These findings challenge the effect of Coccinellini predators on the biological control of non-aphid pests and suggest the possibility of non-target attacks by so-called specialists.

## Background

Ladybird (Coleoptera, Coccinellidae) is a group with diverse feeding habits. Most of them are specialist feeders, while some can have a broad spectrum of prey. The tribe Coccinellini is a monophyletic group comprising 90 genera and over 1000 recognised species worldwide [1]. The Coccinellini are mostly aphidophagous, but their diet is diverse and includes other hemipterous insects (heteropterans, coccids, psyllids, whiteflies), beetle and moth larvae, pollen, fungi or even plant tissue [1–3]. Many species of this tribe are well known as biological control agents, but some subsequently became large scale invaders displacing native ladybirds and causing other environmental problems with non-target effects [4, 5] (e.g., *Harmonia axyridis*, which is causing problems on a global scale [6]. This species is native to Asia and has been introduced into many countries as a biological control agent of pest insect. As a generalist predator, it poses a threat to biodiversity of introduced areas through competition and predation). In general, experimental evidence revealed that some Coccinellini members can feed on non-aphid prey and develop to the adult stage, but they usually show lower performance than those fed on initial prey [7, 8], indicating a potential adaptive process in their prey shift.

**Fig. 1:**
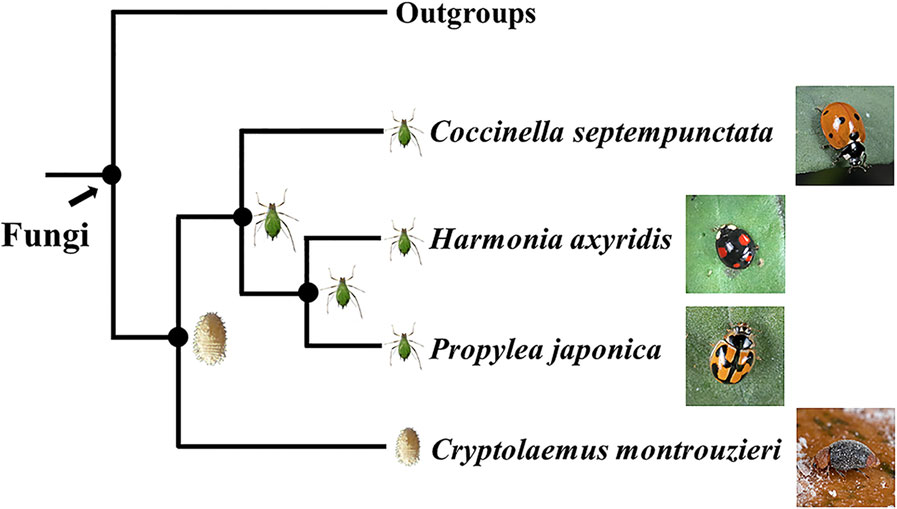
Phylogenetic relationships of the four studied ladybird species and the prey of their common ancestors (adapted from the molecular phylogenetic tree and evolution of food preference of Coccinellidae from Magro et al. [9])

Despite the economic importance of ladybirds, relatively little is known about how they adapt to new prey. The evolution of food preferences suggests that the ancestor of Coccinellini fed on coccids and then became aphidophagous in one transition [3] (Fig. 1). Compared to specialists, generalists should have the possibility and capability of using alternative prey species. Larvae of Coccinellini lost their ancestors’ dorsal defense glands and protective waxes but became more agile and had a higher capacity for predation [1]. According to these morphological and behavioral traits, Coccinellini species could be opportunistic generalists with a variety of prey in their diets. However, it is not clear if these ladybirds adapt well to alternative prey when their primary prey (aphids) are not available.

Transcriptome studies can reveal how insects adapt to novel diets. Recent studies suggest that herbivorous insects use transcriptomic plasticity to their advantage, regulating digestive and detoxifying genes in response to their novel diets [10]. We previously studied the transcriptomic response to novel prey of a coccidophagous ladybird (non-Coccinellini species), *Cryptolaemus montrouzieri* (CM), and we detected reduced performance and differential expression of genes related to biochemical transport, metabolism, and detoxification [11].

To deepen our understanding of the food adaptation of ladybirds, we selected three Coccinellini species, which are commonly used as biological control agents, namely, *Coccinella septempunctata* (CS), *H. axyridis* (HA) and *Propylea japonica* (PJ), as example species. They reportedly feed on prey that include not only aphids but also coccids (mealybugs), whiteflies and psyllids (Table 1). To test if ladybirds perform well in using novel prey, we first compare the life history traits of ladybirds using aphids and mealybugs as the sole food source. To further detect candidate genes related to novel prey adaptation, the transcriptomes of four instar larval stages in two prey treatments were sequenced, and the expression of genes related to development, prey searching, digestion, detoxification and antibacterial activity were analyzed. Finally, the changes in life history traits and gene expression after prey shifts of these three Coccinellini species as well as the previously studied CM were compared. We expected that these Coccinellini species would perform better than CM when using alternative prey.

**Table 1:** Prey that have been reported for *Coccinella septempunctata* (CS), *Harmonia axyridis* (HA), *Propylea japonica* (PJ) and *Cryptolaemus montrouzieri* (CM)

Predator	Prey	Reports
CS	Coccids	Observed in field [12]
	Aphids	Common prey [9]
	Whiteflies	Observed in field [13]
	Psyllids	Tested in lab [14]
HA	Coccids	Observed in field [15]
	Aphids	Common prey [3, 9]
	Whiteflies	Observed in gut content [16]
		Tested in lab [17]
	Psyllids	Observed in field [18]
		Tested in lab [7]
PJ	Coccids	Tested in lab [19]
	Aphids	Common prey [9]
	Whiteflies	Observed in gut content [16]
		Tested in lab [8, 17]
	Psyllids	Tested in lab [20]
CM	Coccids	Common prey [3, 9]
	Aphids	Tested in lab [21, 22]
	Whiteflies	Tested in lab [22]
	Psyllids	Tested in lab [23]

## Results

### Life history traits

**Fig. 2:**
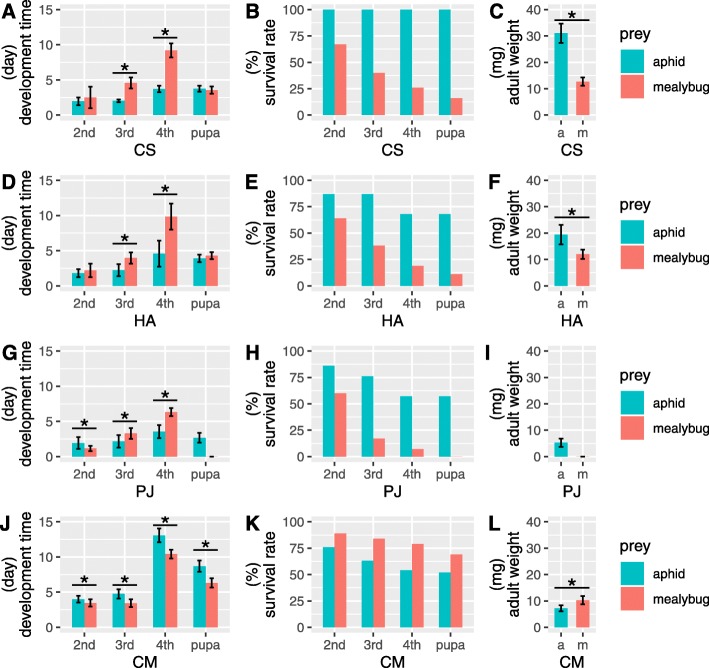
Comparison of life history traits of **a**-**c** *Coccinella septempunctata* (CS), **d**-**f** *Harmonia axyridis* (HA), **g**-**i** *Propylea japonica* (PJ) and **j**-**l** *Cryptolaemus montrouzieri* (CM) in two prey treatments: development time (left panel), survival rate (middle panel) and adult weight (right panel). Asterisks indicate significant differences with *P* < 0.01. Error bars represent standard deviation values

We detected similar patterns of changes in life history traits between CS and HA after shifting their prey from aphids to mealybugs. Both had a significantly longer development time in the 3rd- and 4th-instar larval stages (*P* < 0.01, Fig. 2a and d) in the mealybug treatments. Their survival rates at each stage were lower (Fig. 2b and e), with only 16 and 11% of individuals of CS and HA that fed on mealybugs reaching the adult stage, respectively. Additionally, the weights of newly emerged adults were significantly lower (*P* < 0.01, Fig. 2c and f).

For PJ, the mealybug treatment induced a significantly shorter development time in the 2nd-instar larval stage but a significantly longer development time in the 3rd and 4th instars (*P* < 0.01, Fig. 2g). No individual reached the adult stage when fed mealybugs (Fig. 2h).

According to our previously studied data [11], CM fed the novel prey (aphids) exhibited significantly prolonged larval and pupal development times (*P* < 0.01, Fig. 2j). Their adult weight was significantly lower (*P* < 0.01, Fig. 2l). Although the survival rate in the aphid treatment decreased, more than 50% of individuals developed to the adult stage (Fig. 2k).

### Transcriptional regulation

All raw transcriptome data can be found in the NCBI Short Read Archive (SRA) under BioProject ID PRJNA549114 (BioSample ID: SAMN12071516 - SAMN12071531). We obtained 25–35 million Illumina 125-bp paired-end reads from each of the sequenced samples. De novo transcriptome assemblies created using Trinity resulted in 67,779 (1694), 85,740 (1359), and 50,143 (1899) unigenes (N50) for CS, HA and PJ, respectively (Table 2). The CM transcriptome data in our previous study [11] were also used in the following analyses. After filtering the low expressed genes, more than 70% of genes were annotated in Pfam or NCBI Non-redundant (NR) databases (Table 2). The coefficient of multiple determination (*r*^2^) values calculated within species showed that both of the diet treatments were highly correlated in a group, with a mean *r*^2^ value of 0.919 (see details in Additional file 1: Table S1, S2, S3 and S4), ensuring the biological repeatability. In contrast, the *r*^2^ values between diet treatments were much lower, with a mean value of 0.574.

**Table 2:** Procedures and results of transcriptome filtering, annotation and differential expression

Procedure	CS	HA	PJ	CM
Initial	66,779	85,740	50,143	73,655
Exclude FPKM< 1	8773	9048	9562	9061
Annotated in Pfam	6724	6841	7097	6432
Annotated in NR	7844	8079	8179	7944
Annotated in KOG	5434	6245	5660	6432
Annotated in GO	2987	3055	1707	3176
Up-regulated	294	102	99	534
Down-regulated	202	200	91	257

*FPKM* Fragments per kilobase of transcript per million mapped reads, *NR* Non-redundant database, *KOG* Eukaryotic Orthologous Groups database, *GO* Gene Ontology database

**Fig. 3:**
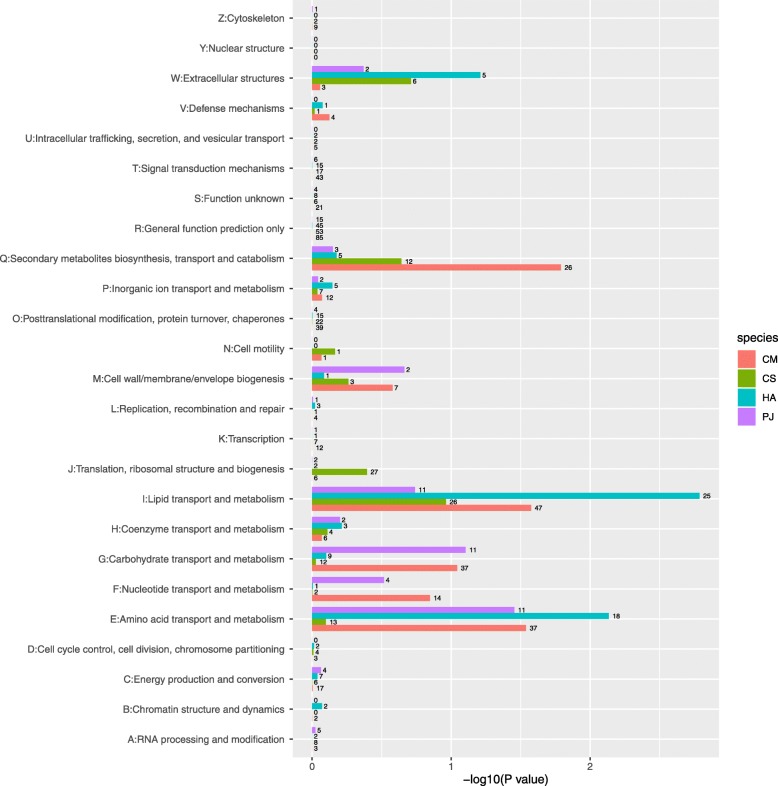
Eukaryotic Orthologous Gene (KOG) enrichment analysis of differentially expressed genes (DEGs) of *Coccinella septempunctata* (CS), *Harmonia axyridis* (HA), *Propylea japonica* (PJ) and *Cryptolaemus montrouzieri* (CM) in two prey treatments. Number of DEGs were in each KOG term was marked on the bar. DEGs with *P* value < 0.05 were considered as significantly enriched

The gene expression of the three Coccinellini species was affected by diet, with the number of DEGs ranging from 190 to 496 (Table 2). PJ had the minimum number of DEGs (99 up-regulated and 91 down-regulated genes), even though no individuals of this species developed to the adult stage. A diet shift in CM led to many more DEGs (534 up-regulated and 257 down-regulated genes). The result of Eukaryotic Orthologous Groups (KOG) annotation of these DEGs showed that most were enriched in E: amino acid, G: carbohydrate and I: lipid transport and metabolism and Q: Secondary metabolites biosynthesis, transport and catabolism (Fig. 3). The result of Gene Ontology (GO) analysis showed that these DEGs mainly enriched in Biological Process (Additional file 1: Table S5).

**Fig. 4:**
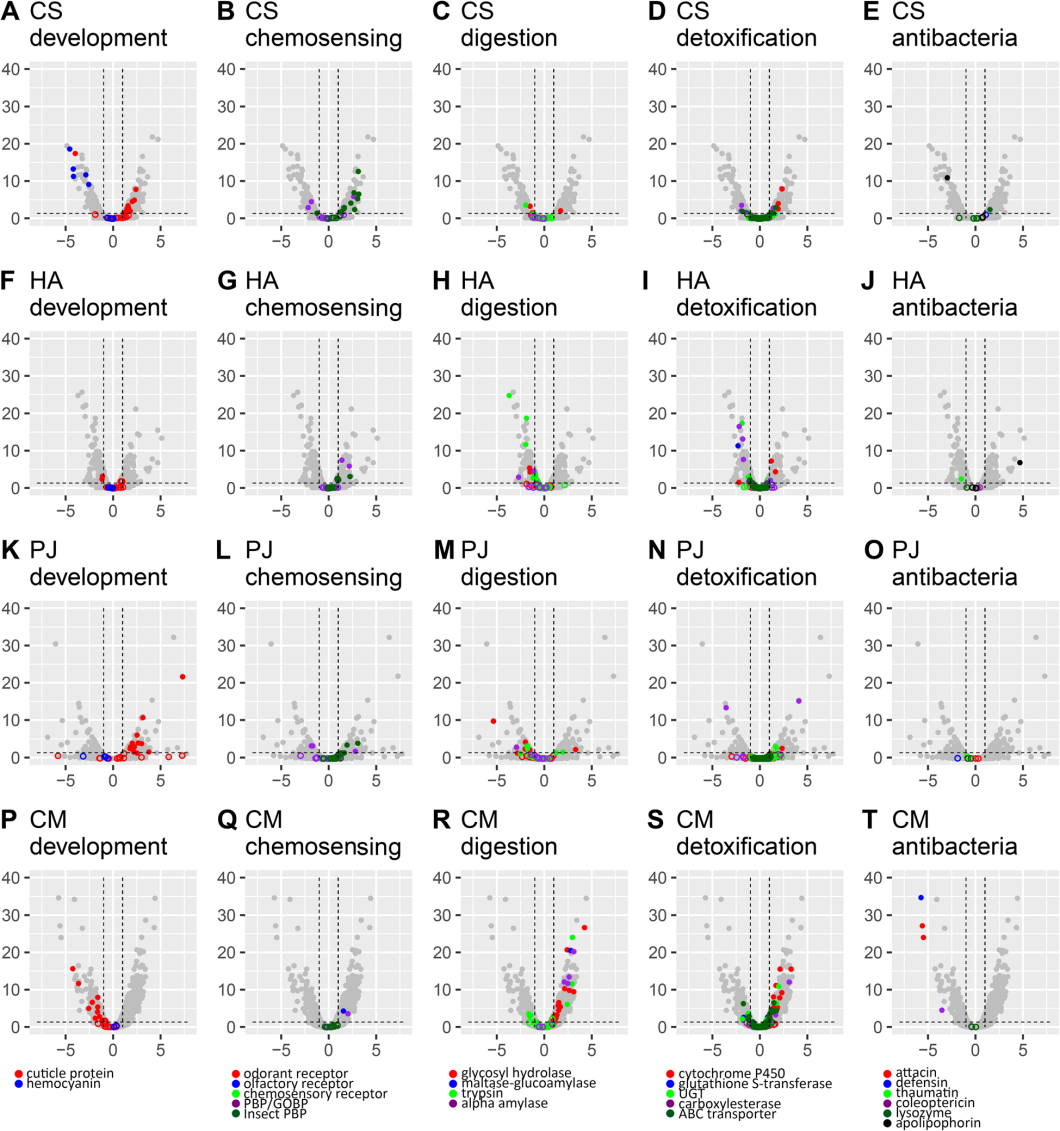
Analyses of differentially expressed genes (DEGs) of **a**-**e** *Coccinella septempunctata* (CS), **f**-**j** *Harmonia axyridis* (HA), **k**-**o** *Propylea japonica* (PJ) and **p**-**t** *Cryptolaemus montrouzieri* (CM) in two prey treatments. DEGs were identified using volcano plots. Horizontal coordinate: log_2_(fold change), vertical coordinate: -log10(Q-value). The studied genes were coloured, and those DEGs were marked as solid circle. Information of Pfam accession of genes can be found in Additional file 1: Table S7. The raw transcriptome data of CM were from our previous study [11]

Among the top 20 genes with the greatest significant absolute log_2_ fold change, we found genes related to development and genes encoding chemosensory proteins, digestive/detoxifying enzymes and antibacterial proteins, which are hypothetically related to adaptation to novel diets (Additional file 2: Table S6). These genes were identified based on Pfam annotation (Additional file 1: Table S7) and mainly classified based on Gilbert et al. [24] and Vilcinskas et al. [25]. Regarding the expression patterns of development-related gene family members, the DEGs of CS, PJ and CM were significantly enriched in cuticle protein genes (Table 3). CS and PJ had a large number of up-regulated DEGs associated with cuticle protein (9 up-regulated genes and 1 down-regulated gene in CS and 13 up-regulated genes in PJ, Fig. 4a and k), while CM had many down-regulated DEGs (10 down-regulated genes, Fig. 4p). We also found the DEGs of CS were significantly enriched in hemocyanin genes (Table 3), with 5 of them were down-regulated (Fig. 4a). The three species had many DEGs related to chemosensing (12 up-regulated and 3 down-regulated genes in CS, 4 up-regulated genes in HA and 5 up-regulated and 2 down-regulated genes in PJ, Fig. 4b, g and l), while only two DEGs were up-regulated in CM (Fig. 4q). Among them, the DEGs of CS and PJ were significantly enriched in Insect PBP (Table 3). With regard to specific genes encoding digestive enzymes, we detected 4 DEGs in CS, 9 in HA, 13 in PJ and 29 in CM (Fig. 4c, h, m and r). We detected a large number of up-regulated DEGs involved in detoxification in CS (14) and CM (32) (Fig. 4d and s). Finally, we detected several DEGs associated with antibacterial activity, but only one in CS and one in HA were up-regulated (Fig. 4e and j). None of antibacterial DEGs was significantly enriched (Table 3).

**Table 3:** Function of the studied genes, their accession in Pfam database and result of enrichment analysis of their differentially expressed genes (DEGs) of *Coccinella septempunctata* (CS), *Harmonia axyridis* (HA), *Propylea japonica* (PJ) and *Cryptolaemus montrouzieri* (CM) in two prey treatments. DEGs with Q value < 0.05 were considered as significantly enriched

Function	Genes	Pfam accession	SP	*P* value	Q value
Development	cuticle protein	PF00379	CS	< 0.001	< 0.001
			HA	0.320	0.754
			PJ	< 0.001	< 0.001
			CM	< 0.001	0.003
	hemocyanin	PF00372, PF03722, PF03723	CS	< 0.001	0.005
			HA	1	1
			PJ	1	1
			CM	1	1
Chemosensing	ordorant receptor	PF02949	CS	1	1
			HA	1	1
			PJ	1	1
			CM	1	1
	olfactory receptor	PF13853, PF14778	CS	1	1
			HA	1	1
			PJ	1	1
			CM	0.325	0.754
	chemosensory receptor	PF08395	CS	1	1
			HA	1	1
			PJ	1	1
			CM	1	1
	PBP/GOBP	PF01395	CS	0.002	0.014
			HA	0.253	0.655
			PJ	0.025	0.139
			CM	0.481	1
	Insect PBP	PF03392	CS	< 0.001	< 0.001
			HA	0.069	0.277
			PJ	0.002	0.014
			CM	1	1
Digestion	glycosyl hydrolase	PF00232, PF00723, PF00722, PF00704, PF00703, PF00728, PF02302, PF01055, PF01301, PF17677, PF07748, PF18438, PF18230, PF01074, PF01532, PF03200, PF02324, PF03662	CS	0.655	1
			HA	0.644	1
			PJ	0.001	0.012
			CM	< 0.001	0.002
	maltase-glucoamylase	PF16863	CS	1	1
			HA	1	1
			PJ	1	1
			CM	0.123	0.410
	trypsin	PF00089	CS	0.927	1
			HA	0.033	0.162
			PJ	0.004	0.027
			CM	0.130	0.410
	alpha amylase	PF00128, PF02806	CS	1	1
			HA	0.057	0.239
			PJ	0.258	0.655
			CM	0.001	0.012
Detoxification	cytochrome P450	PF00067	CS	0.086	0.327
			HA	0.027	0.141
			PJ	0.690	1
			CM	< 0.001	0.002
	glutathione S-transferase	PF02798, PF13409, PF13417, PF00043, PF14497, PF14834, PF13410, PF16865, PF17171, PF17172	CS	0.749	1
			HA	0.536	1
			PJ	1	1
			CM	0.698	1
	UGT	PF00201	CS	0.660	1
			HA	0.145	0.424
			PJ	0.261	0.655
			CM	0.094	0.344
	carboxylesterase	PF00135	CS	0.007	0.042
			HA	0.003	0.021
			PJ	0.231	0.635
			CM	0.040	0.187
	ABC transporter	PF00005, PF00664, PF01061	CS	0.642	1
			HA	0.724	1
			PJ	1	1
			CM	0.340	0.767
Antibacteria	attacin	PF03769	CS	1	1
			HA	1	1
			PJ	1	1
			CM	0.015	0.089
	defensin	PF01097	CS	1	1
			HA	1	1
			PJ	1	1
			CM	0.123	0.410
	thaumatin	PF00314	CS	1	1
			HA	0.044	0.194
			PJ	1	1
			CM	1	1
	lysozyme	PF00062	CS	0.318	0.754
			HA	1	1
			PJ	1	1
			CM	1	1
	apolipophorin	PF07464	CS	0.142	0.424
			HA	0.127	0.410
			PJ	1	1
			CM	1	1
	coleoptericin	PF06286	CS	1	1
			HA	1	1
			PJ	1	1
			CM	0.231	0.635

## Discussion

### Poor performance when fed on alternative prey

In the field, Coccinellini ladybird species have the capability live with alternative prey such as mealybugs, psyllids and whiteflies. Generally, aphidophagous ladybirds are less prey specific than coccidophagous ladybirds [26], demonstrating they potentially have more chances of adaptation to new prey. In this study, we provided mealybugs as the sole prey to three aphidophagous Coccinellini species under laboratory conditions. Compared to individuals fed on aphids, those in the alternative prey treatments exhibited much lower performance, as revealed by the prolongation of development time and decrease in the survival rate and adult weight. Moreover, less than 20% of the individuals fed on alternative prey successfully developed to the adult stage, while up to 50% of the CM individuals succeeded, which was unexpected. Interestingly, CM has long been considered a specialist on mealybugs, based on field observations (it has not been reported to feed on non-mealybug prey in the field), behavior (it requires the wax of mealybugs as an oviposition substrate, and its larvae are much less mobile) [21] and the evolutionary history of its feeding habits (it may not have experienced aphid feeding) [3]. Given the poorer performance in response to a prey shift than in the case of specialist feeding, the classification of these Coccinellini species as generalists is challenged.

### Physiological repsonse to a diet shift

According to the number of DEGs associated with the prey shift, the specialist CM seemed to have a more pronounced physiological response to the alternative prey. In contrast, PJ had the fewest DEGs, but none of the individuals developed into adults. We hypothesized that several shortcomings of prey restricted their use by ladybirds and that ladybirds needed to adapt via transcriptional regulation. When annotating DEGs, we focused on the expression of genes related to development, chemosensing, digestion, detoxification and antibacterial activity, which should be related to the physiology of feeding habits.

We first found up-regulation of cuticle protein genes and down-regulation of hemocyanin genes, mainly in CS and PJ. In the initial stage after insect molting, the cuticle protein expression in fourth instar larvae was high and then decreased after 12 h [27]. Hemocyanin genes, which are associated with the formation of epidermis and nutrient accumulation proteins, are highly expressed in the last-instar larval stage [28, 29]. Thus, the regulation of these genes probably reflected the developmental delay associated with using alternative prey.

Chemosensory proteins are responsible for taste or smell perception and signal transduction. The regulation of genes related to chemosensing is usually involved in changes in food-searching behavior [30–32]. In this study, we detected many DEGs in three Coccinellini species associated with the use of alternative prey, suggesting that these species varied its behavior in searching different prey. In contrast, CM might use one searching strategy for different prey.

Another factor leading to physiological changes in these predators is the differentiation of the chemical composition of mealybugs and aphids [33], which contain different nutrients and toxins. To meet nutritional requirements and cope with toxins accompanying a diet shift, insects usually regulate the expression of genes related to digestion and detoxification [10]. Specifically, inhibition of alpha-amylase and trypsin activity leads to poorer performance in insects [34–36]. Similarly, we found down-regulation of these digestive genes and low performance in Coccinellini species, revealing the effect of prey changes on digestion. We previously detected a large number of up-regulated genes related to detoxification in CM in response to shifting their prey from mealybugs to aphids [11]. In the present study, a prey shift also led to the up-regulation of many detoxifying genes in CS. The up-regulation of detoxifying gene family members has been reported to be associated with host shifts in many herbivorous insects [37–40] and most likely reflects more toxins in novel diets that insects or ladybirds must cope with.

## Conclusions

Our study demonstrated that the use of alternative prey does not well support the development of Coccinellini species. Although these species have a high capacity to be opportunistic generalists, they must adapt to physiological obstacles including chemosensing, digestion and detoxification when using alternative prey. As many Coccinellini species are widely used in pest biological control, our findings have two implications for their optimized applications. First, generalist predators should be less dependent on the availability of one particular prey. However, our findings showed that the absence of initial prey aphids strongly influenced the performance of Coccinellini species. Thus, the strategy of using Coccinellini predators for biological control of non-aphid pests might be unsuitable. Second, the effect of non-target attacks of not only specialists but also so-called generalists should be considered. Future experiments on the effect of more prey on the performance of ladybirds should be conducted in the laboratory and field.

## Methods

### Insect rearing

The three Coccinellini species, namely, CS, HA and PJ, used in this study were maintained with aphids for more than one year (~ 20 generations per year) under laboratory conditions. The citrus mealybug *Planococcus citri* was maintained on fruits of pumpkin (*Cucurbita moschata*). A colony of *Megoura japonica* aphids was reared on plants of the horse bean *Vicia faba*. All insect populations used in this study were from Sun Yat-sen University, Guangzhou, China, and kept in climatic chambers set at 25 ± 1 °C with a relative humidity (RH) of (75 ± 5) % and a photoperiod of 14:10 (L: D) h.

### Experimental diet shift

The larvae of ladybirds fed on mealybugs were tested beginning at the first instar. Since few or none of them survived to the next larval stage, we used second-instar larvae instead. The second-instar larvae from the original populations of CS, HA and PJ were separated into two different lines, which were fed on either aphids or mealybugs. Young larvae of *P. citri* were offered to the candidate ladybirds due to adult mealybugs producing waxes that would influence ladybird prey consumption.

### Comparing life history traits

We investigated the life history traits of ladybirds in the two diet treatments. Development time, survival rate and initial adult emergence (< 24 h) weight were tested, with approximately 50 tested individuals for each diet treatment (CS fed on aphids: 24, mealybugs: 58; HA fed on aphids: 31, mealybugs: 64; PJ fed on aphids: 42, mealybugs: 42).

All life history trait data were analyzed using SPSS 20.0 (SPSS Inc.). All data were first analyzed for adherence to a normal distribution and then subjected to a Kolmogorov-Smirnov test. If the data then fit a normal distribution, they were analyzed using one-way analysis of variance (ANOVA). The means were separated using Tukey tests because Levene’s test indicated homoscedasticity. If the data were not normal distribution, a nonparametric Kruskal-Wallis *H* test was used, followed by a Mann-Whiney *U* test. *P* < 0.05 was considered significant.

### Transcriptional regulation

Fourth-instar larvae (24 h after molting and then 12 h after starvation) from another set of diet treatments were randomly collected for transcriptome sequencing. Two replicates were used for PJ, and three were used for CS and HA. RNA was extracted from the tested individuals before feeding for 24 h after molting and then starvation for 12 h. RNA extraction, RNA-seq analysis, data quality control, de novo assembly and unigene annotation followed Li et al. [11]. We removed the genes showing low expression, i.e., those with a fragments per kilobase of transcript per million mapped reads (FPKM) score < 1, from further analysis. The coefficient of multiple determination (*r*^2^) was calculated between each sample within species. Genes were annotated in Pfam using InterProScan [41], and in NCBI Non-redundant (NR) databases and KOG using BLAST. The regulation of gene expression in each pair of lines was tested using the Bioconductor package DESeq2 [42], with a fold change > 2 or < 0.5 and a false discovery rate (FDR) Q-value < 0.05 used as the criteria for defining differentially expressed genes (DEGs). KOG and Pfam enrichment analysis of DEGs was conducted through a hypergeometric distribution test. Gene Ontology (GO) analysis was performed using the Bioconductor package Clusterprofiler [43].

## Supplementary information

**Additional file 1: Table S1.** Coefficient of multiple determination (*r*^2^) of the *Coccinella septempunctata* transcriptome. CSA: fed on aphids, CSM: fed on mealybugs. **Table S2.** Coefficient of multiple determination (*r*^2^) of the *Harmonia axyridis* transcriptome. HAA: fed on aphids, HAM: fed on mealybugs. **Table S3.** Coefficient of multiple determination (*r*^2^) of the *Propylea japonica* transcriptome. HAA: fed on aphids, HAM: fed on mealybugs. **Table S4**. Coefficient of multiple determination (*r*^2^) of the *Cryptolaemus montrouzieri* transcriptome. CMM: fed on mealybugs, CMA: fed on aphids. **Table S5.** List of significant functionally enriched Gene Ontology (GO) terms. **Table S7** Function of the studied genes and their accession in Pfam database.

**Additional file 2: Table S6.** List of differentially expressed genes (DEGs) in *Coccinella septempunctata* (CS), *Harmonia axyridis* (HA), *Propylea japonica* (PJ) and *Cryptolaemus montrouzieri* (CM) in two prey treatments. Genes are ordered based on the value of log_2_ (fold change).

## Data Availability

The data sets supporting the results of this article are included within the article and its additional files.
