# Supplementary material for: Changes in life history traits and transcriptional regulation of Coccinellini ladybirds in using alternative prey
Source: BMC Genomics. 2020 Jan 14;21:44. doi: 10.1186/s12864-020-6452-0 (PMC6958754; doi:10.1186/s12864-020-6452-0)
Supplement: Supplementary file 1 — Additional file 1: Table S1. Coefficient of multiple determination (r2) of the Coccinella septempunctata transcriptome. CSA: fed on aphids, CSM: fed on mealybugs. Table S2. Coefficient of multiple determination (r2) of the Harmonia axyridis transcriptome. HAA: fed on aphids, HAM: fed on mealybugs. Table S3. Coefficient of multiple determination (r2) of the Propylea japonica transcriptome. HAA: fed on aphids, HAM: fed on mealybugs. Table S4. Coefficient of multiple determination (r2) of the Cryptolaemus montrouzieri transcriptome. CMM: fed on mealybugs, CMA: fed on aphids. Table S5. List of significant functionally enriched Gene Ontology (GO) terms. Table S7 Function of the studied genes and their accession in Pfam database. [file 12864_2020_6452_MOESM1_ESM.docx]

Table S1 Coefficient of multiple determination (*r*^2^) of the *Coccinella septempunctata* transcriptome. CSA: fed on aphids, CSM: fed on mealybugs.

| Sample | CSA1 | CSA2 | CSA3 | CSM1 | CSM2 | CSM3 |
| --- | --- | --- | --- | --- | --- | --- |
| CSA1 |  |  |  |  |  |  |
| CSA2 | 0.981 |  |  |  |  |  |
| CSA3 | 0.993 | 0.989 |  |  |  |  |
| CSM1 | 0.527 | 0.605 | 0.588 |  |  |  |
| CSM2 | 0.491 | 0.606 | 0.557 | 0.835 |  |  |
| CSM3 | 0.413 | 0.521 | 0.476 | 0.787 | 0.984 |  |

Table S2 Coefficient of multiple determination (*r*^2^) of the *Harmonia axyridis* transcriptome. HAA: fed on aphids, HAM: fed on mealybugs.

| Sample | HAA1 | HAA2 | HAA3 | HAM1 | HAM2 | HAM3 |
| --- | --- | --- | --- | --- | --- | --- |
| HAA1 |  |  |  |  |  |  |
| HAA2 | 0.909 |  |  |  |  |  |
| HAA3 | 0.945 | 0.938 |  |  |  |  |
| HAM1 | 0.756 | 0.558 | 0.630 |  |  |  |
| HAM2 | 0.629 | 0.502 | 0.520 | 0.829 |  |  |
| HAM3 | 0.639 | 0.492 | 0.536 | 0.848 | 0.954 |  |

Table S3 Coefficient of multiple determination (*r*^2^) of the *Propylea japonica* transcriptome. HAA: fed on aphids, HAM: fed on mealybugs.

| Sample | PJA1 | PJA2 | PJM1 | PJM2 |
| --- | --- | --- | --- | --- |
| PJA1 |  |  |  |  |
| PJA2 | 0.866 |  |  |  |
| PJM1 | 0.604 | 0.806 |  |  |
| PJM2 | 0.673 | 0.879 | 0.952 |  |

Table S4 Coefficient of multiple determination (*r*^2^) of the *Cryptolaemus montrouzieri* transcriptome. CMM: fed on mealybugs, CMA: fed on aphids.

| Sample | CMM1 | CMM2 | CMA1 | CMA2 |
| --- | --- | --- | --- | --- |
| CMM1 |  |  |  |  |
| CMM2 | 0.961 |  |  |  |
| CMA1 | 0.569 | 0.421 |  |  |
| CMA2 | 0.460 | 0.323 | 0.931 |  |

Table S5 List of significant functionally enriched Gene Ontology (GO) terms. Up or down-regulated genes with GO terms were compared to the total annotated genes. Four tested species included CS: *Coccinella septempunctata*, HA: *Harmonia axyridis*, PJ: *Propylea japonica* and CM: *Cryptolaemus montrouzieri*. Three GO class included BP: biological process, CC: cellular component and MF: molecular function.

| species | Regulate | Class | GO term | Input/reference | Q-value |
| --- | --- | --- | --- | --- | --- |
| CS | up | BP | cellular macromolecule biosynthetic process | 30/476 | <0.001 |
|  |  |  | macromolecule biosynthetic process | 30/481 | <0.001 |
|  |  |  | biosynthetic process | 36/637 | <0.001 |
|  |  |  | cellular biosynthetic process | 35/612 | <0.001 |
|  |  |  | organic substance biosynthetic process | 35/616 | <0.001 |
|  |  |  | cellular nitrogen compound biosynthetic process | 30/504 | <0.001 |
|  |  |  | protein metabolic process | 35/674 | <0.001 |
|  |  |  | gene expression | 29/525 | <0.001 |
|  |  |  | cellular protein metabolic process | 31/592 | 0.001 |
|  |  | CC | cytoplasmic part | 29/444 | <0.001 |
|  |  |  | macromolecular complex | 31/511 | <0.001 |
|  |  |  | small ribosomal subunit | 5/19 | <0.001 |
|  |  |  | cytosolic ribosome | 4/19 | 0.003 |
|  |  |  | cytosolic part | 4/21 | 0.004 |
|  |  |  | cytoplasm | 30/593 | 0.004 |
|  |  |  | large ribosomal subunit | 4/23 | 0.006 |
|  | down | MF | sulfuric ester hydrolase activity | 2/2 | <0.001 |
|  |  | CC | extracellular region | 6/46 | <0.001 |
| HA | down | BP | oxidation-reduction process | 15/205 | <0.001 |
|  |  |  | alpha-amino acid metabolic process | 6/31 | 0.002 |
|  |  |  | isocitrate metabolic process | 3/4 | 0.002 |
|  |  |  | carboxylic acid metabolic process | 11/138 | 0.002 |
|  |  |  | single-organism catabolic process | 7/51 | 0.002 |
|  |  |  | organic acid metabolic process | 11/142 | 0.002 |
|  |  |  | oxoacid metabolic process | 11/142 | 0.002 |
|  |  |  | small molecule metabolic process | 15/263 | 0.002 |
|  |  |  | cellular catabolic process | 8/89 | 0.006 |
|  |  |  | organic acid catabolic process | 4/18 | 0.007 |
|  |  |  | carboxylic acid catabolic process | 4/18 | 0.007 |
|  |  |  | valine metabolic process | 2/2 | 0.007 |
|  |  |  | cellular ketone body metabolic process | 2/2 | 0.007 |
|  |  |  | ketone body catabolic process | 2/2 | 0.007 |
|  |  |  | ketone body metabolic process | 2/2 | 0.007 |
|  |  |  | organonitrogen compound catabolic process | 4/21 | 0.010 |
|  |  |  | single-organism metabolic process | 21/565 | 0.010 |
|  |  |  | alpha-amino acid catabolic process | 3/10 | 0.012 |
|  |  |  | aromatic amino acid family catabolic process | 2/3 | 0.016 |
|  |  |  | branched-chain amino acid metabolic process | 2/3 | 0.016 |
|  |  |  | organic substance catabolic process | 8/116 | 0.017 |
|  |  |  | catabolic process | 8/120 | 0.021 |
|  |  |  | cellular amino acid catabolic process | 3/13 | 0.021 |
|  |  |  | tricarboxylic acid cycle | 3/16 | 0.036 |
|  |  |  | citrate metabolic process | 3/16 | 0.036 |
|  |  |  | cellular amino acid metabolic process | 6/78 | 0.036 |
|  |  |  | cellular biogenic amine metabolic process | 2/5 | 0.036 |
|  |  |  | amine metabolic process | 2/5 | 0.036 |
|  |  |  | cellular amine metabolic process | 2/5 | 0.036 |
|  |  |  | tricarboxylic acid metabolic process | 3/17 | 0.036 |
|  |  |  | energy derivation by oxidation of organic compounds | 4/35 | 0.038 |
|  |  | MF | catalytic activity | 58/1691 | <0.001 |
|  |  |  | oxidoreductase activity, acting on the CH-OH group of donors, NAD or NADP as acceptor | 6/24 | 0.001 |
|  |  |  | isocitrate dehydrogenase (NADP+) activity | 3/4 | 0.002 |
|  |  |  | NAD binding | 5/19 | 0.002 |
|  |  |  | oxidoreductase activity, acting on CH-OH group of donors | 6/32 | 0.003 |
|  |  |  | oxidoreductase activity | 16/252 | 0.005 |
|  |  |  | peptidase activity | 13/181 | 0.005 |
|  |  |  | isocitrate dehydrogenase activity | 3/6 | 0.005 |
|  |  |  | 3-oxoacid CoA-transferase activity | 2/2 | 0.010 |
|  |  |  | hydrolase activity | 27/644 | 0.014 |
|  |  |  | CoA-transferase activity | 2/3 | 0.025 |
|  |  |  | coenzyme binding | 6/59 | 0.032 |
|  |  |  | cofactor binding | 7/82 | 0.036 |
| PJ | up | BP | ribosome biogenesis | 4/40 | 0.045 |
|  |  |  | ribonucleoprotein complex biogenesis | 4/53 | 0.045 |
|  |  |  | rRNA processing | 3/23 | 0.045 |
|  |  |  | rRNA metabolic process | 3/23 | 0.045 |
|  |  | MF | oxidoreductase activity | 6/127 | 0.020 |
|  |  |  | monooxygenase activity | 2/5 | 0.020 |
|  | down | BP | cellular amino acid metabolic process | 6/54 | 0.001 |
|  |  |  | carboxylic acid metabolic process | 6/91 | 0.005 |
|  |  |  | small molecule metabolic process | 8/184 | 0.005 |
|  |  |  | organonitrogen compound metabolic process | 9/243 | 0.005 |
|  |  |  | cellular amino acid biosynthetic process | 3/12 | 0.005 |
|  |  |  | organic acid metabolic process | 6/98 | 0.005 |
|  |  |  | oxoacid metabolic process | 6/98 | 0.005 |
|  |  |  | IMP biosynthetic process | 2/3 | 0.005 |
|  |  |  | 'de novo' IMP biosynthetic process | 2/3 | 0.005 |
|  |  |  | IMP metabolic process | 2/3 | 0.005 |
|  |  |  | purine nucleobase metabolic process | 2/4 | 0.008 |
|  |  |  | organic acid biosynthetic process | 3/18 | 0.008 |
|  |  |  | carboxylic acid biosynthetic process | 3/18 | 0.008 |
|  |  |  | alpha-amino acid metabolic process | 3/22 | 0.014 |
|  |  |  | nucleobase metabolic process | 2/7 | 0.024 |
|  |  |  | single-organism biosynthetic process | 5/102 | 0.024 |
|  |  |  | cellular modified amino acid metabolic process | 2/9 | 0.036 |
|  |  |  | small molecule biosynthetic process | 3/33 | 0.037 |
|  |  |  | purine nucleoside monophosphate biosynthetic process | 2/11 | 0.046 |
|  |  |  | purine ribonucleoside monophosphate biosynthetic process | 2/11 | 0.046 |
|  |  |  | single-organism metabolic process | 9/398 | 0.049 |
|  |  |  | purine-containing compound metabolic process | 3/39 | 0.049 |
| CM | up | BP | purine nucleobase biosynthetic process | 4/5 | 0.005 |
|  |  |  | purine nucleotide biosynthetic process | 10/42 | 0.005 |
|  |  |  | purine nucleoside monophosphate biosynthetic process | 8/28 | 0.005 |
|  |  |  | purine ribonucleoside monophosphate biosynthetic process | 8/28 | 0.005 |
|  |  |  | purine-containing compound biosynthetic process | 10/46 | 0.007 |
|  |  |  | ribonucleoside monophosphate biosynthetic process | 8/31 | 0.009 |
|  |  |  | purine nucleobase metabolic process | 4/7 | 0.010 |
|  |  |  | pigment biosynthetic process | 5/12 | 0.010 |
|  |  |  | purine ribonucleotide biosynthetic process | 9/41 | 0.010 |
|  |  |  | nucleoside monophosphate biosynthetic process | 8/33 | 0.010 |
|  |  |  | pigment metabolic process | 5/13 | 0.013 |
|  |  |  | ribonucleotide biosynthetic process | 9/45 | 0.016 |
|  |  |  | ribose phosphate biosynthetic process | 9/45 | 0.016 |
|  |  |  | nucleobase biosynthetic process | 4/9 | 0.022 |
|  |  |  | nucleotide biosynthetic process | 10/63 | 0.046 |
|  |  |  | nucleobase metabolic process | 4/11 | 0.047 |
|  |  |  | nucleoside phosphate biosynthetic process | 10/64 | 0.047 |
|  |  | MF | hydrolase activity, hydrolyzing O-glycosyl compounds | 13/48 | >0.001 |
|  |  |  | oxidoreductase activity | 33/273 | 0.001 |
|  |  |  | hydrolase activity | 61/710 | 0.010 |
|  | down | BP | small molecule biosynthetic process | 9/55 | >0.001 |
|  |  |  | lipid biosynthetic process | 8/43 | >0.001 |
|  |  |  | organic acid biosynthetic process | 8/44 | >0.001 |
|  |  |  | carboxylic acid biosynthetic process | 8/44 | >0.001 |
|  |  |  | small molecule metabolic process | 16/290 | 0.019 |
|  |  | CC | integral component of membrane | 31/753 | >0.001 |
|  |  |  | intrinsic component of membrane | 31/753 | >0.001 |
|  |  |  | membrane | 39/1201 | 0.001 |
|  |  |  | membrane part | 31/833 | 0.001 |

Table S7 Function of the studied genes and their accession in Pfam database.

| Function | Genes | Pfam accession |
| --- | --- | --- |
| Development | cuticle protein | PF00379 |
|  | hemocyanin | PF00372, PF03722, PF03723 |
| Chemosensing | ordorant receptor | PF02949 |
|  | olfactory receptor | PF13853, PF14778 |
|  | chemosensory receptor | PF08395 |
|  | PBP/GOBP | [PF01395](http://pfam.xfam.org/family/PF01395) |
|  | Insect PBP | PF03392 |
| Digestion | glycosyl hydrolase | PF00232, PF00723, PF00722, PF00704, PF00703, PF00728, PF02302, PF01055, PF01301, PF17677, PF07748, PF18438, PF18230, PF01074, PF01532, PF03200, PF02324, PF03662 |
|  | maltase-glucoamylase | PF16863 |
|  | trypsin | PF00089 |
|  | alpha amylase | PF00128, PF02806 |
| Detoxification | cytochrome P450 | PF00067 |
|  | glutathione S-transferase | PF02798, PF13409, PF13417, PF00043, PF14497, PF14834, PF13410, PF16865, PF17171, PF17172 |
|  | UGT | PF00201 |
|  | carboxylesterase | PF00135 |
|  | ABC transporter | PF00005, PF00664, PF01061 |
| Antibacteria | attacin | PF03769 |
|  | defensin | PF01097 |
|  | thaumatin | PF00314 |
|  | lysozyme | PF00062 |
|  | apolipophorin | PF07464 |
